# *Aspergillus flavus* bZIP-Type Transcription Factors as Promising Novel Targets for Future Aflatoxin Control Strategies

**DOI:** 10.3390/jof12070532

**Published:** 2026-07-19

**Authors:** Ágnes Kata Mondok, Tünde Pusztahelyi, Szilvia Kovács, Barbara Brendzsák, Tamás Emri, István Pócsi, Éva Leiter

**Affiliations:** 1 Department of Molecular Biotechnology and Microbiology, Faculty of Science and Technology, University of Debrecen, H-4010 Debrecen, Hungary; 2 Doctoral School of Pharmaceutical Sciences, University of Debrecen, H-4010 Debrecen, Hungary; 3 Central Laboratory, Faculty of Agricultural and Food Sciences and Environmental Management, University of Debrecen, H-4010 Debrecen, Hungary; 4 Doctoral School of Nutrition and Food Sciences, University of Debrecen, H-4010 Debrecen, Hungary; 5 HUN-REN-UD Fungal Stress Biology Research Group, University of Debrecen, H-4010 Debrecen, Hungary; 6 Food Chain Safety Laboratory Directorate, National Food Chain Safety Office, H-1024 Budapest, Hungary

**Keywords:** *Aspergillus flavus*, bZIP-type transcription factors, environmental stress, fungicide stress, aflatoxin

## Abstract

The bZIP type transcription factors (bZIPs) are global regulators governing vegetative growth, development, stress defense and secondary metabolism, including mycotoxin production in filamentous fungi. In this work, we constructed and phenotypically characterized gene deletion and complementation mutants of some bZIPs, including *Afap1*, *AflatfA*, *LziP*, *AflatfB* and *bZIP6* in *Aspergillus flavus*. Environmental and fungicide stress responses, as well as aflatoxin production of the mutants in both surface cultures and infected maize kernels, were studied. Phenotypic characterization of the mutants revealed that *Afap1* and *AflatfA* were involved in the oxidative (H_2_O_2_, menadione, *tert*-butyl hydroperoxide), cell wall integrity (Congo Red) and heavy metal (CdCl_2_) stress responses of *A. flavus.* In addition, the *Afap1* and *AflatfA* gene deletions decreased the diamide and prothioconazole tolerances of the fungus, respectively. The *ΔLziP* strain showed increased growth in the presence of diamide, while reduced colony diameters were observed after exposure to CdCl_2_ and fludioxonil. The *ΔAflatfB* gene deletion mutant was also sensitive to *t*BOOH, while azoxystrobin and prothioconazole fungicides significantly inhibited the growth of *ΔbZIP6*. When aflatoxin (AFB1) production was measured in surface cultures, decreased AFB1 levels were detected only in the *ΔAflatfA* gene deletion mutant strain. However, in corn kernel infection assays, the *ΔAfap1*, *ΔAflatfA*, and *ΔAflatfB* mutants were characterized by significantly reduced aflatoxin production, while the deletion of *bZIP6* almost completely abolished AFB1 biosynthesis. Our results suggest that *Afap1* and *AflatfA* appear to be promising targets for the development of new antifungal agents, as their inhibition may increase the sensitivity of *A. flavus* to environmental stress, simultaneously reducing the aflatoxin production of the fungus and the use of azoles (*AflatfA*) in the antifungal protection of maize. In addition, *bZIP6* may also be considered as an attractive target for further studies when aiming to eliminate aflatoxin production and minimize the use of azoxystrobin and prothioconazole in maize crop protection.

## 1. Introduction

Basic leucine zipper (bZIP) domain transcription factors (bZIPs) are widespread in fungi and affect nearly all aspects of fungal life, including vegetative growth, asexual and sexual sporulation, environmental stress defense, secondary metabolite production, as well as virulence of pathogenic fungi with various lifestyles [1]. bZIPs evolved from a single ancestral gene, and the diversification of the bZIP superfamily was closely linked to the emergence of multicellular lineages [2]. Not surprisingly, bZIPs have become essential components of global regulatory networks that fine-tune cell proliferation and defense against environmental stress [2]. In the baker’s yeast *Saccharomyces cerevisiae*, a family of eight bZIP-type transcription factors could be identified, which is called the Yap (yeast activator protein) transcription factor family. Among them, Yap1 plays an important role in defense against oxidative stress, while Yap2 helps yeast cells develop cadmium resistance when its gene is overexpressed [3]. In the fission yeast *Schizosaccharomyces pombe*, Pap1, which is the ortholog of *S. cerevisiae* Yap1, and another bZIP transcription factor, Atf1, which is the ortholog of human Atf2, play important roles in regulating defense against various types of environmental stress [4,5]. In the filamentous model fungus *Aspergillus nidulans*, the Yap1 ortholog NapA and the Atf1 ortholog AtfA are key players in the orchestration of oxidative stress defense and the biosynthesis of sterigmatocystin, a precursor of aflatoxins [6,7,8,9]. A recent combined ChIP-sequencing and RNA-sequencing study revealed that *A. nidulans* AtfA is a master integrator that synchronizes asexual development, adaptation to environmental stress, and the metabolic preparedness that underlies them [10,11].

Environmental stress, and especially oxidative stress, has been shown to activate secondary metabolite gene clusters, leading to the production of both valuable pharmaceutical raw materials and highly harmful mycotoxins [12,13,14,15,16,17]. The abundance of such observations led to the formulation of the “oxidative stress theory of mycotoxin biosynthesis” [12]. Not surprisingly, bZIPs, as master regulators of oxidative stress defense, are closely linked to the regulation of mycotoxin biosynthesis in various mycotoxin-producing fungi through the modulation of intracellular reactive oxygen species (ROS) levels, including *Aspergillus parasiticus* (aflatoxins, ApYapA; [18]), *Aspergillus ochraceus* (ochratoxin A, AoYap1; [19]) and *Aspergillus nidulans* (sterigmatocystin, NapA; [7]). Other bZIPs have been shown to tightly and directly co-regulate oxidative stress response and mycotoxin production in *A. parasiticus* (aflatoxins, AtfB; [20,21]), *Aspergillus flavus* (aflatoxins, AflAtfA and AflAtfB; [22,23]), *Fusarium verticillioides* (fumonisins, FvAtfA; [24]) and *A. nidulans* (sterigmatocystin, AtfA; [9]).

In a previous substantial study, Zhao et al. (2022) [23] functionally characterized 15 bZIPs of *A. flavus via* generating gene deletion strains and screening for vegetative growth, sexual and asexual development, aflatoxin production, environmental stress sensitivity (oxidative stress—H_2_O_2_, cell wall stress—Calcofluor White/CFW, osmotic stress—sorbitol, acid stress—pH 5.0, alkali stress—pH 9.0) and pathogenicity (on maize kernels) phenotypes. It is important to note that only the *ΔMetR* gene deletion strain was genetically complemented in that study, and neither additional environmental stress conditions, including various oxidative and heavy metal stress exposures, nor fungicide tolerances were examined (Zhao et al. 2022) [23].

To fill these knowledge gaps and based on data from the previous literature on bZIP orthologs well-characterized in other fungi, we selected five *A. flavus* bZIP transcription factor genes for a more detailed functional study, with particular attention to environmental stress and fungicide tolerances, as well as aflatoxin production in maize grains. The selected bZIP genes included *Afap1* (locus ID: AFLA_012763), *AflatfA* (AFLA_001005), *LziP* (AFLA_003889), *AflatfB* (AFLA_009637; [23]) and *bZIP6* (AFLA_014249; [23]). We constructed a full set of gene deletion and genetically complemented strains, and oxidative stress was initiated using H_2_O_2_ (increases intracellular peroxide levels), *tert*-butyl hydroperoxide (*t*BOOH, intensifies lipid peroxidation reactions), menadione sodium bisulfite (MSB, elevates intracellular superoxide radical anion—O_2_^●−^—levels) and diamide (stoichometrically oxidizes glutathione and protein thiols to disulfides) [7,11,24,25]. Cell integrity and heavy metal stresses were generated by Congo Red dye and CdCl_2_, respectively [9,26]. In addition, we also tested the growth inhibitory effects of three broad-spectrum fungicides with different modes of action, namely fludioxonil (a phenylpyrrole, which improperly activates HogA MAPK signaling; [27,28]), azoxystrobin (interferes with mitochondrial respiration; [28]) and prothioconazole (a C14-demethylase inhibitor in sterol biosynthesis; [28]), which are frequently used in agriculture against plant pathogenic fungi. Concomitantly, we also tested the aflatoxin production of wild-type control and bZIP gene deletion strains on potato dextrose agar (PDA) supplemented with (NH_4_)_2_SO_4_ and maize kernels.

With these data in our hands, we sought to answer the interesting question of whether targeting any of these bZIP-type transcription factors would lead to a general decrease in environmental stress tolerance of *A. flavus* and a concomitant decrease in its aflatoxin production. Such bZIPs would be attractive and promising targets in future fungicide research as well as in the development of new pre-harvest technologies that prevent the formation of harmful aflatoxins and thus their entry into the feed and food chain.

## 2. Materials and Methods

### 2.1. Strains and Culture Conditions 

*A. flavus* strains summarized in Table S1 were maintained on Czapek–Dox medium (30 g of sucrose, 3 g of NaNO_3_, 1 g of K_2_HPO_4_, 0.5 g of KCl, 0.5 g of MgSO_4_·7H_2_0, 0.01 g of FeSO_4_, 20 g of agar in 1 L) [29] supplemented with 10 mM (NH_4_)_2_SO_4_ and 50 μg/mL uracil, 5 mM uridine as required, and were incubated at 30 °C for 6 d [30]. Conidia harvested from these 6 d old plates were used in all further experiments.

### 2.2. Construction of Gene Deletion and Complemented Strains

Gene deletion mutants were constructed by the Double-Joint PCR method [30,31,32] with primers listed in Table S2. The amplified deletion cassettes carrying pyrithiamine-resistant marker were used to transform protoplasts of the *A. flavus* SRRC1713 (AF70) strain using the Extralyse lysing enzyme [33]. Single-copy transformants were selected after PCR analysis [31]. To generate complemented strains, genes with their putative promoter and terminator sequences were amplified and fused with *A. nidulans pyrG* with the primers presented in Table S2. The complementation cassettes were directly used to transform the corresponding gene deletion mutants as described previously [33]. The genotypes of the complemented strains were verified by PCR, confirming the integration of the promoter–gene–terminator sequence into the genome of the corresponding gene deletion mutants.

### 2.3. Environmental Stress and Fungicide Sensitivity Studies 

Phenotypical characterization of the *A. flavus* SRRC1713 (AF70) control, the bZIP gene deletion and genetically complemented strains was carried out on Glucose Minimal Medium (GMM) agar plates. In each experiment, 5 µL aliquots of 2 × 10^6^/mL spore suspension were point-inoculated onto (NH_4_)_2_SO_4_ and uracil–uridine supplemented GMM (0.52 g MgSO_4_·7H_2_O, 1.52 g KH_2_PO_4_, 10 g of glucose, 0.52 g KCl, 1 mL trace element solution and 18 g agar in 1 L; [34]) and the cultures were incubated at 30 °C for 5 d. The growth inhibitory effects of the following stress generating agents and fungicides were tested at the concentrations indicated in parentheses: H_2_O_2_ (2.5 mM), MSB (0.1 mM), *t*BOOH (0.25 mM), diamide (1.5 mM), Congo Red (300 µg/mL), CdCl_2_ (0.25 mM), fludioxonil (0.5 µg/mL), azoxystrobin (10 µg/mL), and prothioconazole (5 µg/mL). The concentrations of the tested compounds were carefully selected so that no or negligible growth was observed with the *ΔAfap1* gene deletion strain in the presence of molecules that induce oxidative stress, while in the case of other chemicals, concentrations were optimized and chosen based on data from the previous literature [35,36,37,38].

### 2.4. Aflatoxin Measurements

To determine aflatoxin production of the strains, PDA (potato dextrose agar) medium supplemented with 10 mM (NH_4_)_2_SO_4_ and 50 μg/mL uracil, 5 mM uridine as required, was inoculated with 1 × 10^4^ spores (in 5 μL aliquots) harvested after 6 d incubation. PDA plates were incubated for 5 d at 30 °C and then stored at −20 °C until further analysis. 

To recover aflatoxins from PDA plates containing *A. flavus* colonies grown on their surfaces, the mass of each plate was measured, then the plates were macerated in a sterile Stomacher homogenizer bag with NaCl (1 g/10 g sample) and 20% water–80% methanol solution (2 mL solution/1 g sample) with a Stomacher machine. Aflatoxin was purified using the AflaTest aflatoxin immunoaffinity column. Immuno-purified aflatoxin was then concentrated by an evaporator and measured by HPLC as described previously [39]. In addition, Biopure Aflatoxin B1 analytical standard solution was applied to the column. The relative standard deviation, as the absolute value of the coefficient of variation, was calculated and found to be below 10% in all cases [40].

To determine aflatoxin production by the tested *A. flavus* strains in maize kernels, we used *Zea mays* B73 hybrid strain, which exhibits high sensitivity to plant-pathogenic filamentous fungal species like *A. flavus* [41]. The kernels were soaked in distilled water for one hour and then autoclaved at 121 °C for 20 min to disinfect and kill them. The grains (14 g per experiment) were then wounded at one point with a sterile scalpel to create an infection site and then soaked overnight (typically 12 h) in a spore solution (1 × 10^6^ spores/30 mL) of the tested *A. flavus* strain on a rocking table (Bio-Rad UltraRocker, Hercules, CA, USA) at 120 rpm. The infected grains were then transferred onto a sterile, wet filter paper, which was placed in a glass Petri dish and incubated at 30 °C for 21 d [42].

To determine aflatoxin concentration, infected grain samples were ground into powder, then 12.5 g per sample was mixed with 1.25 g of NaCl, suspended in 25 mL of 20% water–80% methanol solution, and stirred on a magnetic stirrer for 10 min. Aflatoxin concentrations were measured by HPLC as described above.

### 2.5. Statistical Analysis

All experiments were conducted in three independent sets, and mean ± SD values were calculated and are presented. Significance of the difference between the treated and untreated cultures in each strain and between the strains in each treatment, as well as the interaction between the effect of the strain and treatment were studied by simultaneous tests [43]. *p*-values less than 5% were considered statistically significant. In the statistical analysis of the aflatoxin measurements, statistical significances were calculated using Student’s *t*-test, and *p*-values less than 5% were considered statistically significant.

## 3. Results

### 3.1. Construction and Genotyping of the Gene Deletion and Complementation Mutants 

In this study, we successfully constructed a series of bZIP gene deletion mutants and their genetically complemented strains. The following genes were included in the study: *Afap1* (locus ID: AFLA_012763), *AflatfA* (AFLA_001005), *LziP* (AFLA_003889, [23]), *AflatfB* (AFLA_009637; [23]), which is also called *atfC* [40], and *bZIP6* (AFLA_014249; [23]) following the protocol of Cary et al. (2017) [30]. As shown in Figures S1–S6, homokaryotic null mutants were generated in *A. flavus* for each bZIP gene using the pyrithiamine resistance selection marker [30]. First, we demonstrated the absence of the given bZIP gene using gene-specific primers, and then we confirmed the homologous integration of the gene deletion cassette by PCR with external primers and the restriction digestion pattern of the PCR products (Figures S1–S6 and Table S2; [31]). bZIP gene complemented strains were also constructed using the *A. nidulans pyrG* marker to complement the *pyrG* mutation in the *A. flavus* deletion mutants (Figure S7). Again, appropriate reintroduction of the given bZIP gene was checked by PCR (Figure S1).

### 3.2. Environmental Stress Sensitivity Phenotypes of the bZIP Mutants

**Figure 1:**
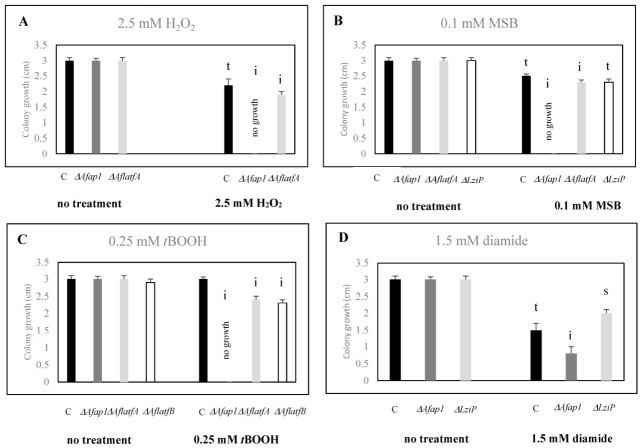
Colony growth (in cm) of the control and mutants in the presence of oxidative stress-generating agents ((**A**): 2.5 mM H_2_O_2_, (**B**): 0.1 mM MSB, (**C**): 0.25 mM *t*BOOH, (**D**): 1.5 mM diamide). C: control (SRRC1713) strain. Data are presented as mean ± SD values calculated from three independent experiments. Significance of the difference between the treated and untreated cultures (t) in each strain and between the strains in each treatment (s), as well as the interaction between the effect of the strain and treatment (i), was studied by Simultaneous tests [43]. Only significant changes in colony growth are presented.

**Figure 2:**
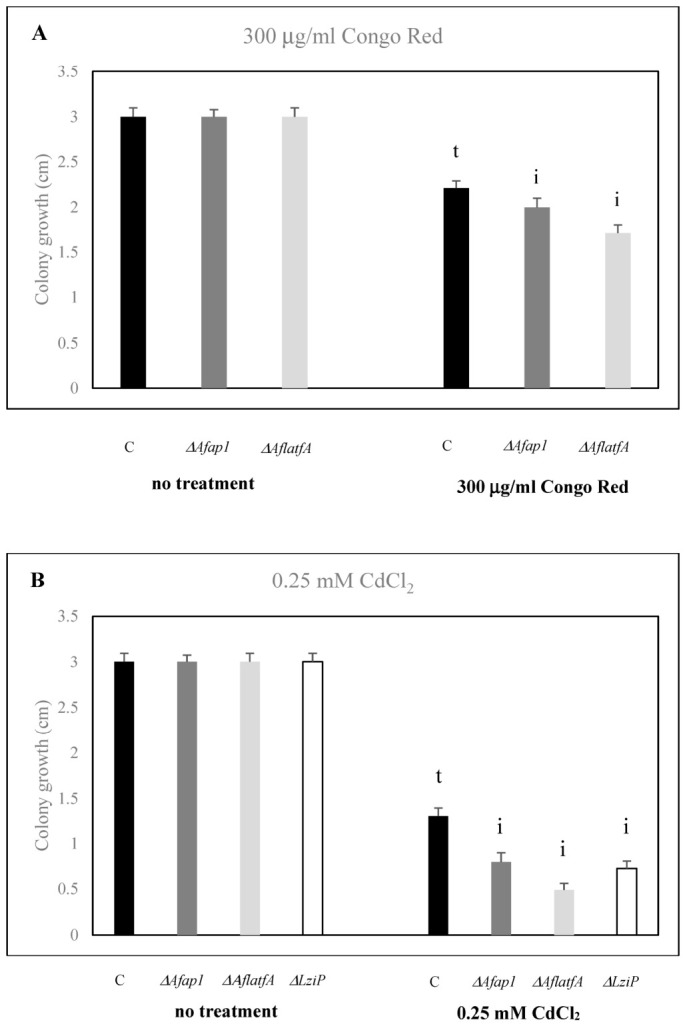
Colony growth (in cm) of the control and mutants in the presence of cell wall integrity (300 μg/mL Congo Red) (**A**) and heavy metal stress (0.25 mM CdCl_2_) (**B**) generating agents. C: control (SRRC1713) strain. Data are presented as mean ± SD values calculated from three independent experiments. Significance of the difference between the treated and untreated cultures (t) in each strain and between the strains in each treatment (s), as well as the interaction between the effect of the strain and treatment (i), was studied by simultaneous tests [43]. Only significant changes in colony growth are presented.

Some bZIP gene deletion mutants showed increased environmental stress sensitivities, which phenotypes were successfully reversed in the genetically complemented strains (Figure 1, Figure 2 and Figure S8, Table 1). For example, reduced growth phenotypes were observed in GMM agar cultures of bZIP mutants exposed to various oxidative stress inducers (mutant strains with increased sensitivities are shown in parentheses) as follows: 2.5 mM H_2_O_2_ (*ΔAfap1* and *ΔAtfA*), 2.5 mM *t*BOOH (*ΔAfap1*, *ΔAflatfA* and *ΔAflatfB*), 0.1 mM MSB (*ΔAfap1* and *ΔAflatfA*) and 1.5 mM diamide (*ΔAfap1*) (Figure 1 and Figure S8, Table 1). It is worth noting that the growth of *ΔAfap1* was completely inhibited in the presence of 2.5 mM H_2_O_2_, 0.1 mM MSB and 2.5 mM *t*BOOH; meanwhile, the *ΔLziP* strain showed increased growth in the presence of diamide (Figure 1 and Figure S8, Table 1). It is important to highlight that the *ΔAfap1* and *ΔAflatfA* strains were also sensitive to 300 μg/mL Congo Red treatment, which induces cell wall integrity stress, and the *ΔAfap1, ΔAflatfA* and *ΔLziP* gene deletion mutants were less tolerant to heavy metal stress induced by CdCl_2_ (Figure 2 and Figure S8, Table 1).

### 3.3. Fungicide Sensitivity of the bZIP Mutants

**Figure 3:**
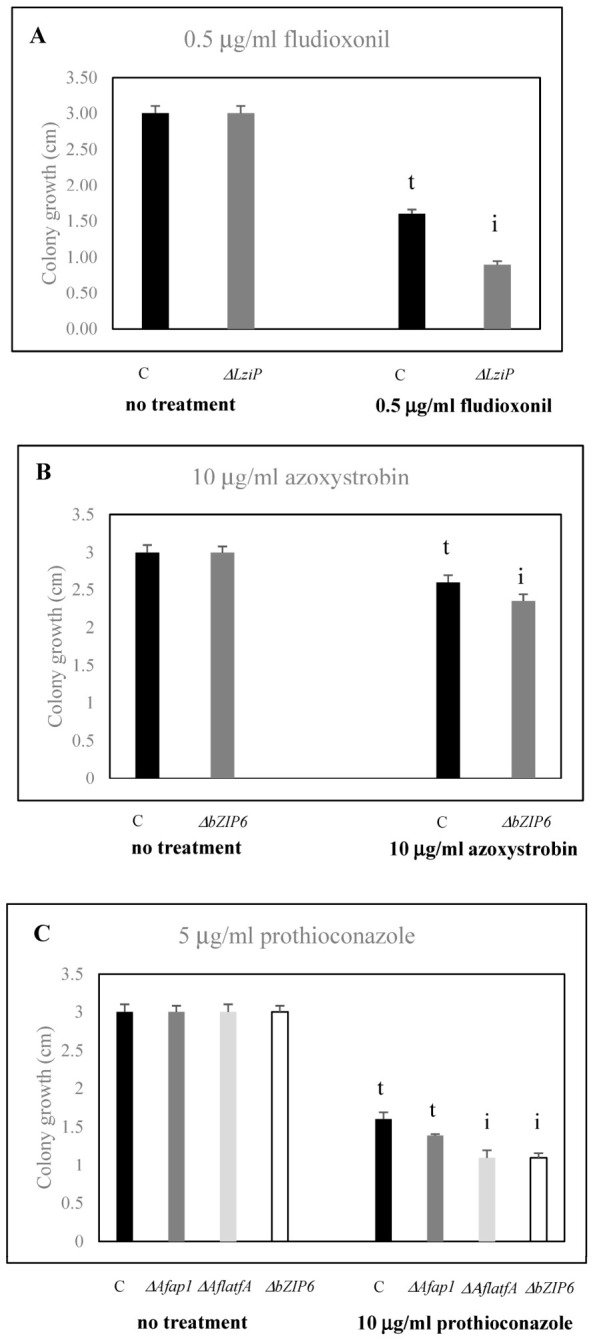
Colony growth (in cm) of the control and mutants in the presence of fungicides (**A**): 0.5 μg/mL fludioxonil, (**B**): 10 μg/mL azoxystrobin, (**C**): 5 μg/mL prothioconazole. C: control (SRRC1713) strain. Data are presented as mean ± SD values calculated from three independent experiments. Significance of the difference between the treated and untreated cultures (t) in each strain and between the strains in each treatment (s), as well as the interaction between the effect of the strain and treatment (i), was studied by simultaneous tests [43]. Only significant changes in colony growth are presented.

We also mapped the antifungal susceptibility of the gene deletion mutants in the presence of some frequently used fungicides. It is noteworthy that 0.5 μg/mL fludioxonil (*ΔLziP*), 10 μg/mL azoxystrobin (*ΔbZIP6*) and 5 μg/mL prothioconazole (*ΔAflatfA* and *ΔbZIP6*) significantly reduced the colony diameters of some bZIP mutants (shown in parentheses) in comparison to the SRRC1713 (AF70) control strains (Figure 3 and Figure S8, Table 1).

### 3.4. Aflatoxin Determination of the Mutants

**Figure 4:**
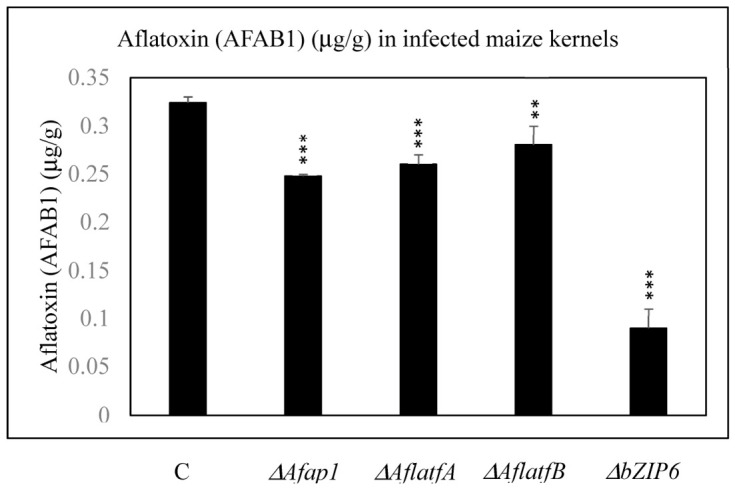
Aflatoxin production in maize kernels of the control and mutants. Data are presented as mean ± SD values calculated from three independent experiments. Significant differences between control and mutant cultures (**, *p* < 1% and ***, *p* < 0.1%) are indicated. Only significant changes in aflatoxin B1 production are presented.

Aflatoxin production of the bZIP gene deletion mutants was quantified in both PDA plate and maize kernel assays (Table 2, Figure 4). Reduced aflatoxin biosynthesis was observed only in the *ΔAflatfA* mutant on PDA medium at a significance level of *p* < 5%; meanwhile, aflatoxin production was significantly lower in the *ΔAfap1*, *ΔAflatfA* and *ΔAflatfB* mutants and nearly abolished in the *ΔbZIP6* strains when measured in infected maize kernels (Table 2, Figure 4). Importantly, all these phenotypes were successfully reversed in genetically complemented strains (Table 2).

## 4. Discussion

### 4.1. Novel bZIP Targets to Decrease Environmental Stress Tolerance by Aspergillus flavus

Climate change is escalating aflatoxin-related food safety problems worldwide, including in Europe [44,45,46,47]. Although the development of new aflatoxin prevention and decontamination technologies is an area of intensive research [47,48,49,50], there is a need for original approaches to effectively tackle aflatoxin-related problems under rapidly changing climatic environmental conditions [47]. Unraveling the global and local transcriptional regulation of aflatoxin biosynthesis is of paramount importance for several reasons, but uncovering the master regulators that synchronize aflatoxin biosynthesis with environmental stress defense seems to be of paramount importance when trying to identify future molecular-level intervention targets [12,17,47]. Attacking and neutralizing these key regulators, for example, with RNA interference-based, host-induced aflatoxin control technologies, seems to be a fairly attractive idea [47,51,52].

Aflatoxins are very dangerous carcinogenic compounds [53], but they also possess antioxidant properties, so they can even be considered natural scavengers of ROS [54], which can enhance the stress tolerance of aflatoxin-producing fungi both under harsh environmental conditions and within host organisms [54,55]. Not surprisingly, increasing evidence supports the idea that aflatoxin biosynthesis is initiated by ROS and that oxidative stress defense and aflatoxin biosynthesis are co-regulated and even share common regulatory elements in aflatoxin-producing fungi [12,13,15,18,19,21,56,57,58,59,60]. We should keep in mind that other types of environmental stresses, such as drought, which is increasingly common in Europe as an obvious consequence of the changing climate, also trigger the formation of ROS and, at the same time, increase aflatoxin production in fungi [44,47,55].

Plant-plant pathogenic fungus interactions can also be perturbed by environmental heavy metals, including CdCl_2_ stresses [61,62], and the cadmium burden may influence aflatoxin production by *A. flavus* in a dose-dependent manner [63]. Co-occurrence of aflatoxin and cadmium in corn grains and flour has been analyzed and is being considered in various countries [64,65,66].

In *A. flavus*, AfAp1, AflAtfA and, to a lesser extent, LZiP bZIPs seem to be the key players in the regulation of various types of environmental stress responses, as indicated in Figure 1 and Figure 2, Table 1. AfAp1 is the ortholog of *S. cerevisiae* Yap1, a master regulator of baker’s yeast defense against oxidative stress [3], and appears to be involved not only in defense against oxidative stress when *A. flavus* is exposed to H_2_O_2_, *t*BOOH, MSB, and diamide, but also in the regulation of cell wall integrity (in the presence of Congo Red) and cadmium stress responses. In good accordance with our observations, Guan et al. (2019) [67] and Zhao et al. (2022) [23] also reported in their previous studies that H_2_O_2_ significantly inhibited the growth of the *ΔAafap1* mutant. Furthermore, in contrast to what was observed in this study with Congo Red, the deletion of *Afap1* did not affect the growth of *A. flavus* when exposed to Calcofluor White, another cell wall stress-inducing compound, according to Zhao et al. (2022) [23].

Considering other *Aspergillus* species, in *A. nidulans*, the AfAp1 ortholog, NapA, also contributed to the defense against oxidative stress (in H_2_O_2_, MSB and *t*BOOH exposures) [6,7,8]. Furthermore, in the opportunistic human pathogen *A. fumigatus*, AfYap1, the orthologue of AfAp1, was also involved in defense against oxidative stress during H_2_O_2_ and menadiuone exposures, but not against diamide and NO radicals [68]. To our knowledge, this is the first study to test heavy metal stress tolerance in *A. flavus* bZIP gene deletion mutants, and the *ΔAfap1* strain showed high sensitivity to CdCl_2_. Similarly, deletion of the gene encoding the Yap1 ortholog, NcAp-1, in *Neurospora crassa* increased the fungus’s sensitivity to CdCl_2_ [69].

Another key regulator of general environmental stress defense (apart from diamide) in *A. flavus* is AflAtfA, the ortholog of fission yeast Atf1 [4,5] and *A. nidulans* AtfA [9,10,25]. Deletion of *AflatfA* increased the oxidative stress sensitivity of *A. flavus* in the presence of H_2_O_2_, MSB and *t*BOOH (Figure 1 and Table 1), and increased H_2_O_2_ sensitivity was also observed in the previous study of Zhao et al. (2022) [23]. 

Similarly, Atf1-AflAtfA orthologs are key players in the regulation of oxidative stress defense in both vegetative tissues and resting conidia of *A. nidulans* [9,10,11,25,70,71]. Furthermore, conidia of the *A. fumigatus ΔatfA* mutant showed decreased tolerance to oxidative and heat stress [72]. In line with our observations with Congo Red, Zhao et al. (2022) [23] reported an increased cell wall stress sensitivity of the *ΔAflatfA* strain when exposed to Calcofluor White. Considering other fungal pathogens of maize, *F. verticillioides* FvAftfA orchestrates oxidative (H_2_O_2_, MSB) and cell wall integrity (Congo Red) stress defenses similar to *A. flavus* AflatfA. Interestingly, meanwhile, *A. flavus* AflatfA seems to be a key player in the regulation of cadmium stress defense, and the deletion of neither *A. nidulans atfA* [9,26] nor *F. verticilliodes FvatfA* [24] affected the cadmium tolerance of the wild-type strains. Nevertheless, the role of AtfA in cadmium stress defense cannot be excluded in *A. nidulans* either, as it is likely that the remarkably high flexibility of the regulation of the cadmium stress response in this fungus enabled the activation of effective alternative adaptation pathways, which well compensated for the lack of AtfA [26].

In our study, the *ΔAflatfB* mutant was characterized by increased *t*BOOH sensitivity, similar to the observation of Zhao et al. (2022) [23]. In *A. nidulans,* the AFLA_009637 (AflAtfB) ortholog AtfB plays an important role in regulating heat (conidia) and cadmium (vegetative tissue) stress responses [9]. In *A. fumigatus*, AtfB is involved in defense against oxidative stress and cell wall integrity stress (tested with Congo Red dye) [73].

Interestingly, the *A. flavus ΔLziP* mutant showed wild-type tolerance to the peroxides H_2_O_2_, and *t*BOOH and MSB, but was sensitive to CdCl_2_ treatment and, unexpectedly, it was more tolerant to diamide exposure (Figure 1 and Figure 2, Table 1). However, Zhao et al. (2022) [23] previously reported increased H_2_O_2_ sensitivity of their *ΔLziP* gene deletion strain. The function of the LziP orthologous bZIP transcription factor ZipA (AN11891) has been investigated in *A. nidulans* among *Aspergillus* species [6]. Interestingly, in this case, deletion of *zipA* resulted in increased H_2_O_2_ tolerance, while overexpression of *zipA* increased the sensitivity of *A. nidulans* to MSB and diamide elicited oxidative stress [6].

### 4.2. Novel bZIP Targets to Reduce Aflatoxin Production by Aspergillus flavus

As previously reported, fungal bZIPs can modulate ROS-induced mycotoxin production, either indirectly, through upregulation of antioxidant defenses and thereby reducing intracellular ROS levels [7,8,12,18,19], or directly, through synchronized coregulation of oxidative stress defense and mycotoxin biosynthetic cluster genes [1,9,10,20,21,22,23,24,59], which may involve the binding of bZIPs to their promoter binding sites [18,20,21,59].

In the case of *A. parasiticus* (an aflatoxin producer) and *A. nidulans* (a sterigmatocystin producer), Yap1 orthologs appear to reduce mycotoxin production via decreasing intracellular ROS levels [7,8,18]; meanwhile, Atf1-AflAtfA and/or AtfB orthologs are positive regulators of aflatoxin and sterigmatocystin biosynthesis in *A. parasiticus* [20,21,55], *A. flavus* [22,23] and *A. nidulans* [9]. Surprisingly, aflatoxin production was reduced in *ΔAfap1* strains, as reported by Guan et al. (2019) [67] in a mutant strain grown on YES agar plates and also observed in this study where aflatoxin was measured in infected maize kernels (Table 2, Figure 4), while Zhao et al. (2022) [23] detected wild-type levels of aflatoxin in their *ΔAP1* (*ΔAfap1*) strain grown on YES agar. Similar to previous findings with *ΔAflatfA* (*ΔatfA*) and *ΔAflatfB* (*ΔatfB*) by Wang et al. (2022) [22] and Zhao et al. (2022) [23], we also recorded reduced aflatoxin production for the *ΔAflatfA* (on PDA plate and in infected maize kernels) and *ΔAflatfB* (in infected maize kernels) mutants and considerably reduced aflatoxin production for the *ΔbZIP6* strain (in infected maize kernels) (Table 2, Figure 4).

### 4.3. Novel bZIP Targets to Reduce the Use of Fungicides in Corn Protection

Broad-spectrum fungicides like fludioxonil, azoxystrobin and prothioconazole are frequently used in agriculture and were tested with our bZIP gene deletion mutants (Figure 3 and Table 1). Interestingly, only the *ΔLziP* strain showed an increased sensitivity to fludioxonil, which improperly activates the SakA/HogA MAPK signaling pathway [27,28]. It is worth noting that AtfA functions downstream of SakA/HogA MAPK in *A. nidulans* [1,27,71], where they cooperate to regulate oxidative, osmotic, and fludioxonil stress responses [71,74]. Fludioxonil is frequently used in crop seed protection in agriculture to control seed-borne and soil-borne fungal pathogens, including corn seeds [28]. In the control of mycotoxin-producing fungi, for example, the use of fludioxonil to eliminate *Aspergillus carbonarius* infections in vineyards not only reduced fungal growth, but also the production of ochratoxin by the fungus [75]. Furthermore, fludioxonil is also used against the maize pathogen *F. verticilliodes*, but *A. flavus* showed greater sensitivity than *F. verticillioides* both in vitro studies and in the field [37]. 

In the case of azoxystrobin, which is often used to control fungal pathogens of maize, only the *ΔbZIP6* mutant showed increased sensitivity. This fungicide inhibits mitochondrial respiration and ultimately causes starvation stress in fungi [28,75] and has been reported to reduce aflatoxin production by *A. flavus* in in vitro studies [76].

Treatments with prothioconazole, a C14-demethylase inhibitor [28], significantly hindered the growth of two gene deletion strains, *ΔAflatfA* and *ΔbZIP6*. Prothioconazole inhibits ergosterol biosynthesis in fungi and the Yap1 transcription factor coordinates ergosterol biosynthesis in *S. cerevisiae* [77]. Furthermore, putative AtfA binding sites were identified in the promoter of “Squalene-ergosterol pathway” genes in *A. nidulans* by Emri et al. (2021) [26]. Field studies by Masiello et al. (2019) [37] demonstrated that prothioconazole reduced *A. flavus* contamination of maize by 56% and was also most effective against *Fusarium* infections. Interestingly, the Ads-4 ZipA ortholog bZIP transcription factor conferred resistance to azoles in *A. fumigatus* [78].

Targeting bZIPs in maize crop protection would help reduce the amount of fungicides used in agriculture, which is in line with the objectives of the European Green Deal directive, which sets a target of reducing the use and risks of chemical pesticides by 50% by 2030 [79,80,81,82]. The use of lower doses of fungicides in agriculture may also delay the emergence of fungicide resistance in plant pathogenic fungi [83,84,85] and cross-resistance to clinical antifungals in human pathogenic fungi, especially in the case of azoles [86,87,88,89].

## 5. Conclusions

As demonstrated in this study, AfAp1, AflatfA and, to a lesser extent, AflatfB and LziP, appear to be promising targets when future *A. flavus* control technologies aim to weaken the environmental stress defense system of this aflatoxigenic mold. Furthermore, AfAp1, AflatfA, AflatfB and especially bZIP6 seem to be particularly attractive targets when we also want to downregulate aflatoxin biosynthesis in *A. flavus*. In addition, targeting the bZIPs LZiP (fludioxonil), bZIP6 (azoxystrobin, prothioconazole), and AflatfA (prothioconazole) would help us to reduce the use and risks of the chemical fungicides indicated in parentheses. For the development of future antifungal crop protection strategies, the bZIP transcription factor AflatfA appears to be a particularly attractive target, as its inhibition may increase the environmental stress sensitivity and reduce the aflatoxin production of *A. flavus*, while minimizing the use of azoles in maize crop protection. bZIP6, which negatively affects aflatoxin production and fungicide (azoxystrobin and prothioconazole) tolerance, should also be involved in further antimycotic development studies.

## Figures and Tables

**Table 1:** Colony growth diameter (in cm) of the control SRRC1713 and mutant strains in the absence (no treatment) and presence of environmental and fungicide stressors ^1^.

	SRRC1713 (Control)	Δ*Afap1*	*Afap1* Comp	Δ*AflatfA*	*AflatfA* Comp	Δ*LziP*	*LziP* Comp	Δ*AflatfB*	*AflatfB* Comp	Δ*bZIP6*	*bZIP6* Comp
no treatment	3 ± 0.1	3.0 ± 0.08	2.9 ± 0.1	3 ± 0.1	2.9 ± 0.2	3 ± 0.1	3.1 ± 0.103	2.9 ± 0.1	2.97 ± 0.09	3.0 ± 0.08	2.97 ± 0.08
2.5 mM H_2_O_2_	2.2 ± 0.2 **^t^**	no growth **^i^**	2.23 ± 0.05	1.9 ± 0.1 **^i^**	2.20 ± 0.05	2.20 ± 0.05	2.2 ± 0.048	2.3 ± 0.1	2.0 ± 0.08	2.30 ± 0.09	2.1 ± 0.1
0.1 mM MSB	2.50 ± 0.06 **^t^**	no growth **^i^**	2.5 ± 0.1	2.30 ± 0.07 **^i^**	2.4 ± 0.1	2.3 ± 0.1 **^t^**	2.4 ± 0.1	2.6 ± 0.3	2.3 ± 0.2	2.6 ± 0.2	2.50 ± 0.06
0.25 mM *t*BOOH	3.0 ± 0.06	no growth **^i^**	2.8 ± 0.1	2.4 ± 0.1 **^i^**	2.7 ± 0.3	2.8 ± 0.4	2.8 ± 0.2	2.3 ± 0.1 **^i^**	2.9 ± 0.1	2.8 ± 0.2	2.8 ± 0.2
1.5 mM diamide	1.5 ± 0.2 **^t^**	0.8 ± 0.2 **^i^**	1.65 ± 0.06	1.47 ± 0.08	1.70 ± 0.05	2 ± 0.1 **^s^**	1.41 ± 0.01	1.4 ± 0.1	1.4 ± 0.03	1.47 ± 0.06	1.48 ± 0.05
300 μg/mL Congo Red	2.21 ± 0.08 **^t^**	2 ± 0.1 **^i^**	2.19 ± 0.09	1.71 ± 0.09 **^i^**	2.20 ± 0.07	2.2 ± 0.1	2.1 ± 0.1	2.2 ± 0.1	2.1 ± 0.3	2.2 ± 0.2	2.1 ± 0.1
0.25 mM CdCl_2_	1.30 ± 0.09 **^t^**	0.8 ± 0.1 **^i^**	1.4 ± 0.08	0.49 ± 0.07 **^i^**	1.20 ± 0.05	0.73 ± 0.08 **^i^**	1.40 ± 0.08	1.29 ± 0.03	1.40 ± 0.03	1.25 ± 0.06	1.28 ± 0.09
0.5 μg/mL fludioxonil	1.60 ± 0.06 **^t^**	1.49 ± 0.03	1.6 ± 0.1	1.60 ± 0.03	1.70 ± 0.06	0.90 ± 0.05 **^i^**	1.60 ± 0.08	1.44 ± 0.05	1.6 ± 0.1	1.60 ± 0.09	1.6 ± 0.1
10 μg/mL azoxystrobin	2.6 ± 0.1 **^t^**	2.6 ± 0.2	2.40 ± 0.04	2.2 ± 0.3	2.5 ± 0.06	2.4 ± 0.3	2.50 ± 0.06	2.38 ± 0.08	2.4 ± 0.2	2.35 ± 0.09 **^i^**	2.56 ± 0.04
5 μg/mL prothioconazole	1.60 ± 0.09 **^t^**	1.39 ± 0.02 **^t^**	1.7 ± 0.2	1.1 ± 0.1 **^i^**	1.6 ± 0.1	1.40 ± 0.09	1.40 ± 0.05	1.4 ± 0.1	1.4 ± 0.1	1.10 ± 0.06 **^i^**	1.7 ± 0.3

^1^ Data are presented as mean ± SD values calculated from three independent experiments. Significance of the difference between the treated and untreated cultures (**^t^**) in each strain and between the strains in each treatment (**^s^**), as well as the interaction between the effect of the strain and treatment (**^i^**), was studied by simultaneous tests [43].

**Table 2:** Aflatoxin (AFB1) production of the control, deletion and complementation mutants determined on PDA plate and in infected maize kernels.

*A. flavus* Strains	Aflatoxin (AFB1) (ng/g/cm^2^) on PDA Plate ^1,2^	Aflatoxin (AFB1) (μg/g) in Infected Maize Kernels ^1^
SRRC 1713 (control)	3.9 ± 0.6	0.324 ± 0.006
*ΔAfap1*	2.7 ± 0.9	0.248 ± 0.002 ***
*Afap1* comp	3.6 ± 1.2	0.317 ± 8
*ΔAflatfA*	2.8 ± 0.3 *	0.26 ± 0.01 ***
*AflatfA* comp	3.7 ± 1.6	0.321 ± 0.005
*ΔLziP*	4.5 ± 0.2	0.44 ± 0.09
*LziP* comp	4.2 ± 0.9	0.34 ± 0.08
*ΔAflatfB*	4.2 ± 0.6	0.28 ± 0.02 **
*AflatfB* comp	4.2 ± 1.5	0.323 ± 0.004
*ΔbZIP6*	4.2 ± 0.6	0.09 ± 0.02 ***
*bZIP6* comp	3.9 ± 0.9	0.33 ± 0.04

^1^ Data are presented as mean ± SD values calculated from three independent experiments. Significant differences between control and mutant cultures (*, *p* < 5%, **, *p* < 1% and ***, *p* < 0.1%) are indicated. ^2^ Aflatoxin concentration was normalized to agar plate mass and colony area.

## Data Availability

The data that support the findings of this study are available from the corresponding authors upon reasonable request.

## Supplementary Materials

The following supporting information can be downloaded at: https://www.mdpi.com/article/10.3390/jof12070532/s1, Table S1: *Aspergillus* strains used in this study [90,91]. Table S2: oligonucleotides used in this study. Figure S1: Genotypic check of the gene deletion and complementation mutants. Figure S2: Genotypic check of the *ΔAfap1* gene deletion mutant. Figure S3: Genotypic check of the *ΔAflatfA* gene deletion mutant. Figure S4: Genotypic check of the *ΔLziP* gene deletion mutant. Figure S5: Genotypic check of the *ΔAflatfB* gene deletion mutant. Figure S6: Genotypic check of the *ΔbZIP6* gene deletion mutant. Figure S7: Schematic representation of the construction of the bZIPs’ complementation cassette. Figure S8: Stress sensitivity of the control and mutant strains.
