# Supplementary material for: Aspergillus flavus bZIP-Type Transcription Factors as Promising Novel Targets for Future Aflatoxin Control Strategies
Source: J Fungi (Basel). 2026 Jul 19;12(7):532. doi: 10.3390/jof12070532 (PMC13412632; doi:10.3390/jof12070532)
Supplement: Supplementary file 1 [file jof-12-00532-s001.zip › jof-4333994 Figures S1-S8.pdf]

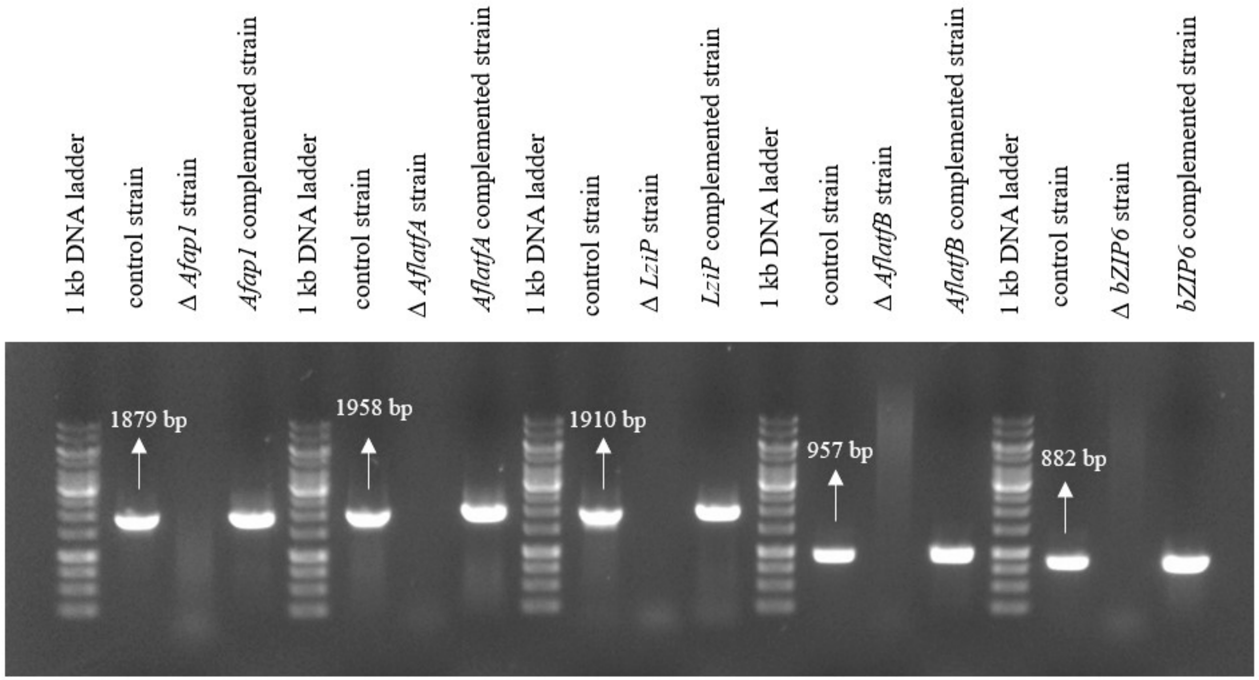

Figure S1 Genotypic check of the gene deletion and complementation mutants. A PCR check of the presence and absence of the relevant bZIP genes with gene specific primers in the control (SRRC1713), deletion and complementation mutants (Table S2).

A

B

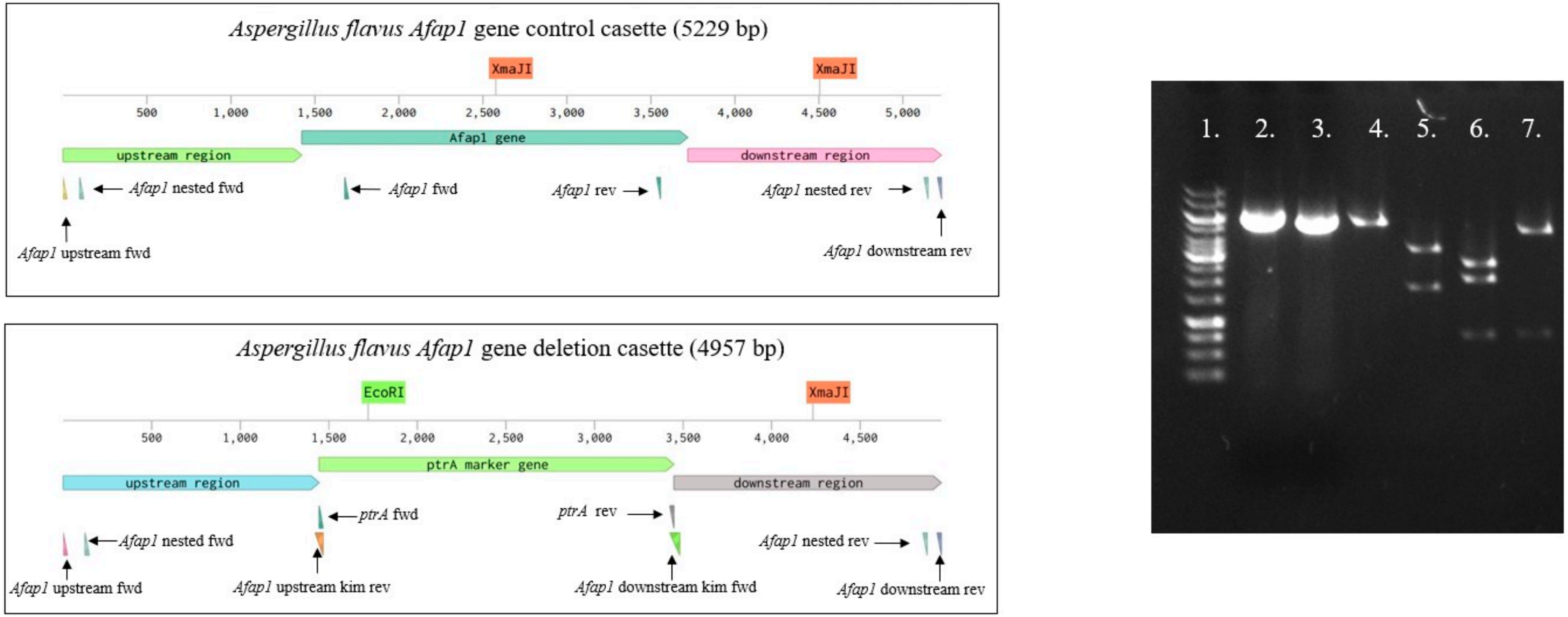

Figure S2 Genotypic check of the *ΔAfap1* gene deletion mutant. PCR check of the gene replacement using external primers (Table S2), following restriction digestion of the amplicons (Yu et al. 2004) [31]. **A** Schematic representation of the control and *Afap1* gene deletion cassettes. Schematic diagrams were retrieved from https://benchling.com. **B** Confirmation of the gene replacement. lanes: **1**. 1kb DNA ladder, **2.** PCR product from control (5229 nt), **3.** PCR product from Δ*Afapa1* (4975 nt), **4.** *EcoRI* restriction digestion of the control PCR product (5229 nt, no cutting), **5.** *EcoRI* restriction digestion of the PCR product of mutant (fragment sizes: 3236 nt, 1721 nt), **6.** *XmaJI* restriction digestion of the control PCR product (fragment sizes: 2576 nt, 1928 nt, 725 nt), **7.** *XmaJI* restriction digestion of the mutant PCR product (fragment sizes: 4232 nt, 725 nt)

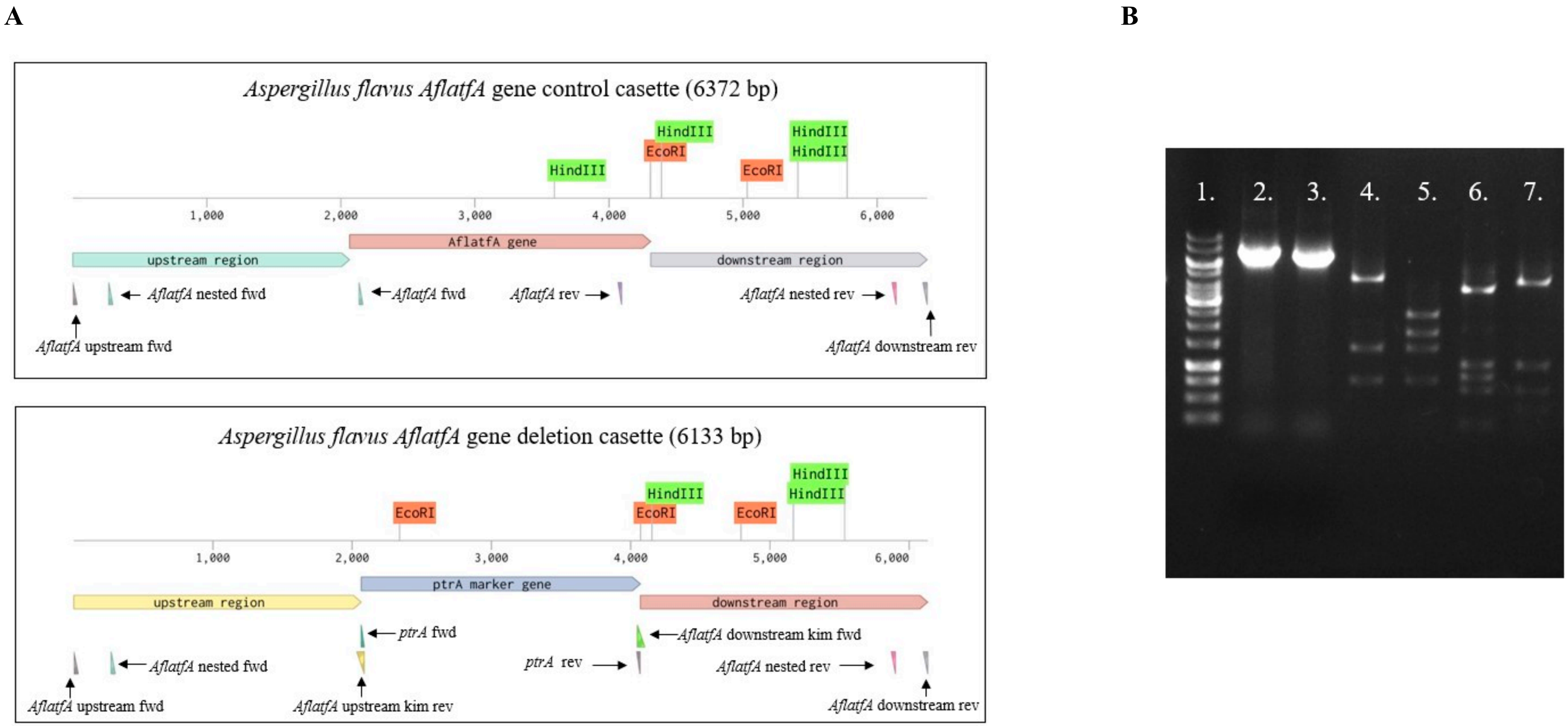

Figure S3 Genotypic check of the *ΔAflatfA* gene deletion mutant. PCR check of the gene replacement using external primers (Table S2), following restriction digestion of the amplicons (Yu et al. 2004) [31]. **A** Schematic representation of the control and *AflatfA* gene deletion cassettes. Schematic diagrams were retrieved from https://benchling.com. **B** Confirmation of the gene replacement. lanes: **1**. 1kb DNA ladder, **2.** PCR product from control (6372 nt), **3.** PCR product from Δ*AflatfA* (6133 nt), **4.** *EcoRI* restriction digestion of the control PCR product (fragment sizes: 4309 nt, 1342 nt, 721 nt), **5.** *EcoRI* restriction digestion of the PCR product of mutant (fragment sizes: 2340 nt, 1730 nt, 1342 nt, 721nt), **6.** *HindIII* restriction digestion of the control PCR product (fragment sizes: 3591 nt, 1016 nt, 800 nt, 597 nt, 365 nt), **7.** *HindIII* restriction digestion of the mutant PCR product (fragment sizes: 4152 nt, 1016 nt, 597 nt, 368 nt)

A B

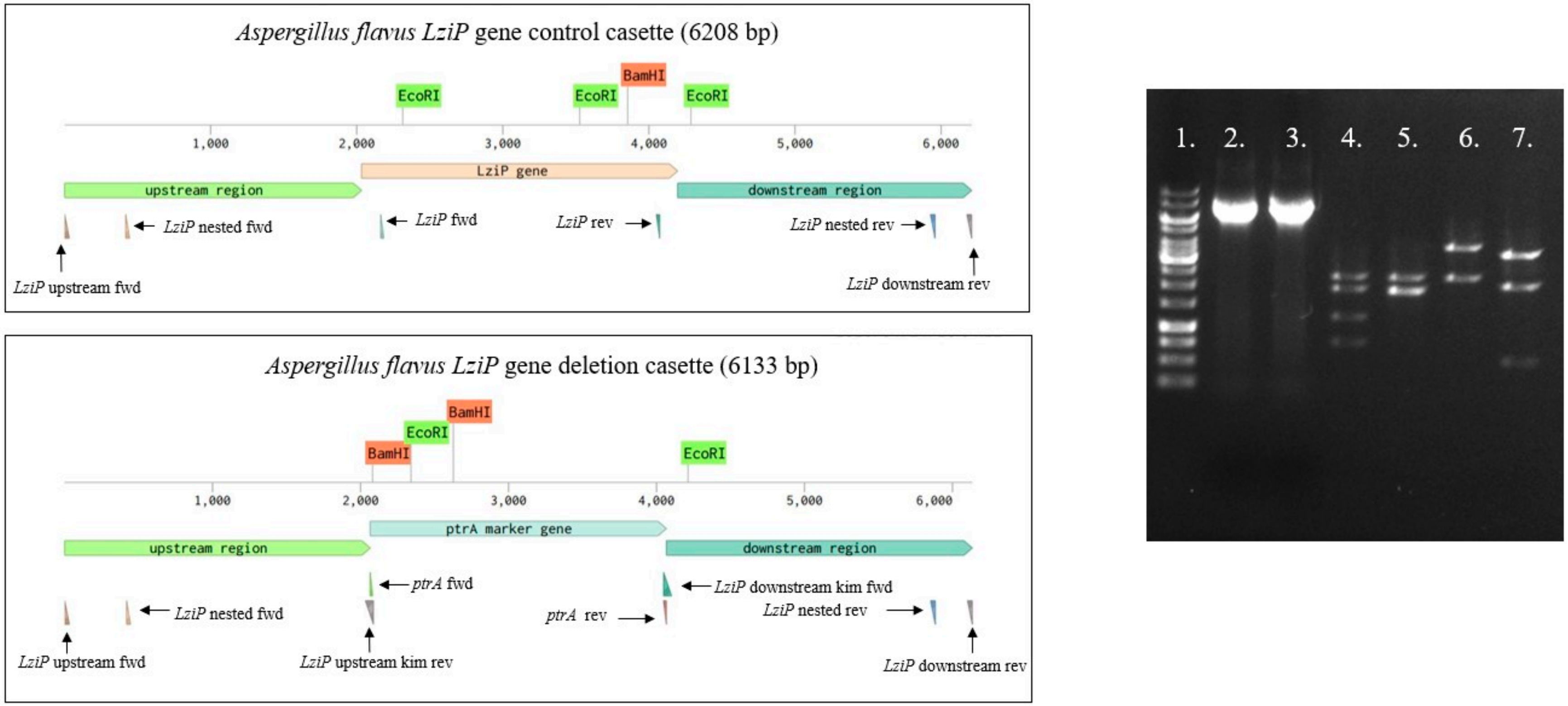

Figure S4 Genotypic check of the *ΔLziP* gene deletion mutant. PCR check of the gene replacement using external primers (Table S2), following restriction digestion of the amplicons (Yu et al. 2004) [31]. **A** Schematic representation of the control and *LziP* gene deletion cassettes. Schematic diagrams were retrieved from https://benchling.com. **B** Confirmation of the gene replacement. lanes: **1**. 1kb DNA ladder, **2.** PCR product from control (6208 nt), **3.** PCR product from Δ*LziP* (6133 nt), **4.** *EcoRI* restriction digestion of the control PCR product (fragment sizes: 2314 nt, 1921 nt, 1211 nt, 762 nt), **5.** *EcoRI* restriction digestion of the PCR product of mutant (fragment sizes: 2340 nt, 1921 nt, 1872 nt), **6.** *BamHI* restriction digestion of the control PCR product (fragment sizes: 3854 nt, 2354 nt), **7.** *BamHI* restriction digestion of the mutant PCR product (fragment sizes: 3507 nt, 2080 nt, 546 nt)

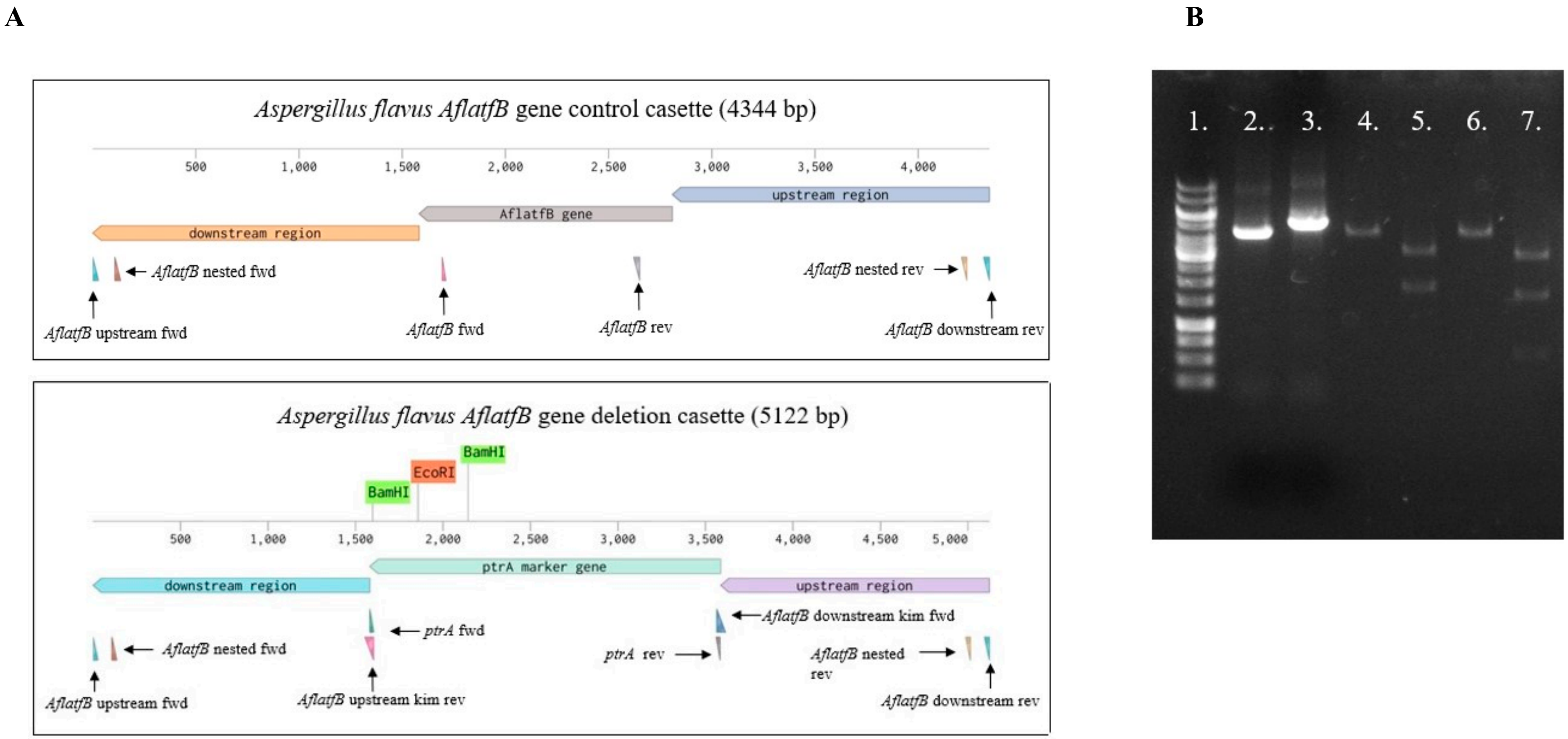

Figure S5 Genotypic check of the *ΔAflatfB* gene deletion mutant. PCR check of the gene replacement using external primers (Table S2), following restriction digestion of the amplicons (Yu et al. 2004) [31]. **A** Schematic representation of the control and *AflatfB* gene deletion cassettes. Schematic diagrams were retrieved from https://benchling.com. **B** Confirmation of the gene replacement. lanes: **1**. 1kb DNA ladder, **2.** PCR product from control (4344 nt), **3.** PCR product from Δ*AflatfB* (5122 nt), **4.** *EcoRI* restriction digestion of the control PCR product (4344 nt, no cutting), **5.** *EcoRI* restriction digestion of the PCR product of mutant (fragment sizes: 3265 nt,1857 nt), **6.** *BamHI* restriction digestion of the control PCR product (4344 nt, no cutting), **7.** *BamHI* restriction digestion of the mutant PCR product (fragment sizes: 2979 nt, 1597 nt, 546 nt)

A

B

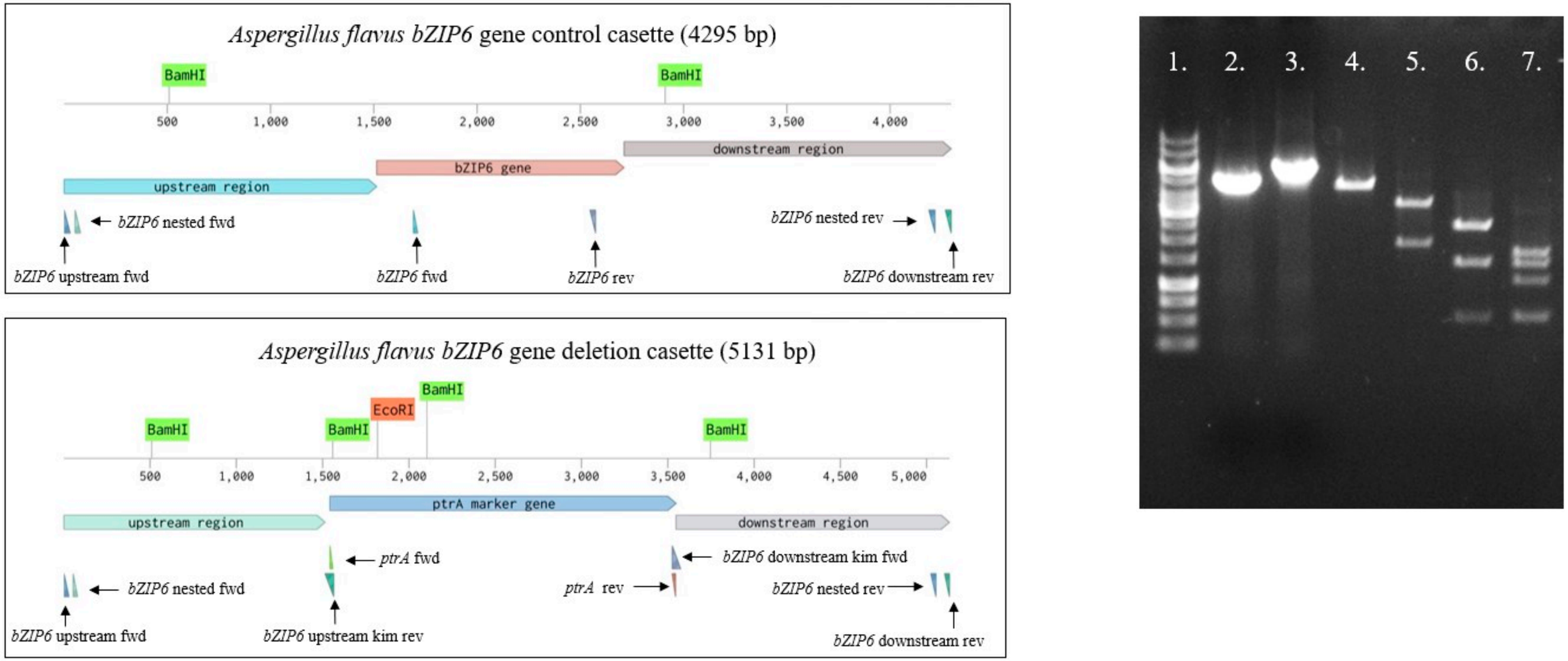

Figure S6 Genotypic check of the *ΔbZIP6* gene deletion mutant. PCR check of the gene replacement using external primers (Table S2), following restriction digestion of the amplicons (Yu et al. 2004) [31]. **A** Schematic representation of the control and *bZIP6* gene deletion cassettes. Schematic diagrams were retrieved from https://benchling.com. **B** Confirmation of the gene replacement. lanes: **1**. 1kb DNA ladder, **2.** PCR product from control (4295 nt), **3.** PCR product from Δ*bZIP6* (5131 nt), **4.** *EcoRI* restriction digestion of the control PCR product (4295 nt, no cutting), **5.** *EcoRI* restriction digestion of the PCR product of mutant (fragment sizes: 3314 nt, 1817 nt), **6.** *BamHI* restriction digestion of the control PCR product (fragment sizes: 2402 nt, 1385 nt, 508 nt), **7.** *BamHI* restriction digestion of the mutant PCR product (fragment sizes: 1643 nt, 1385 nt, 1049 nt)

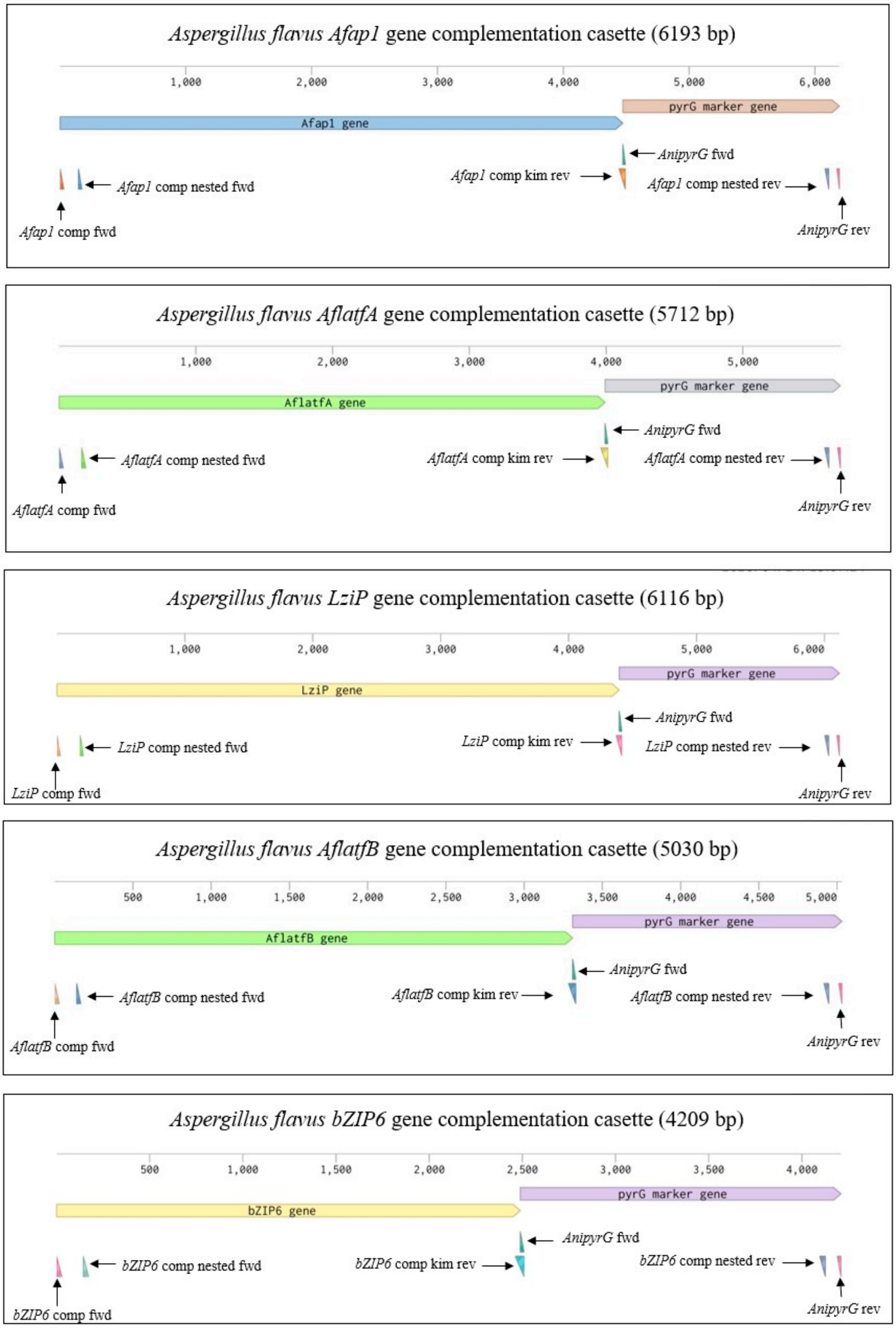

Figure S7 Schematic representation of the construction of the bZIPs' complementation cassette. Schematic diagrams were retrieved from https://benchling.com.

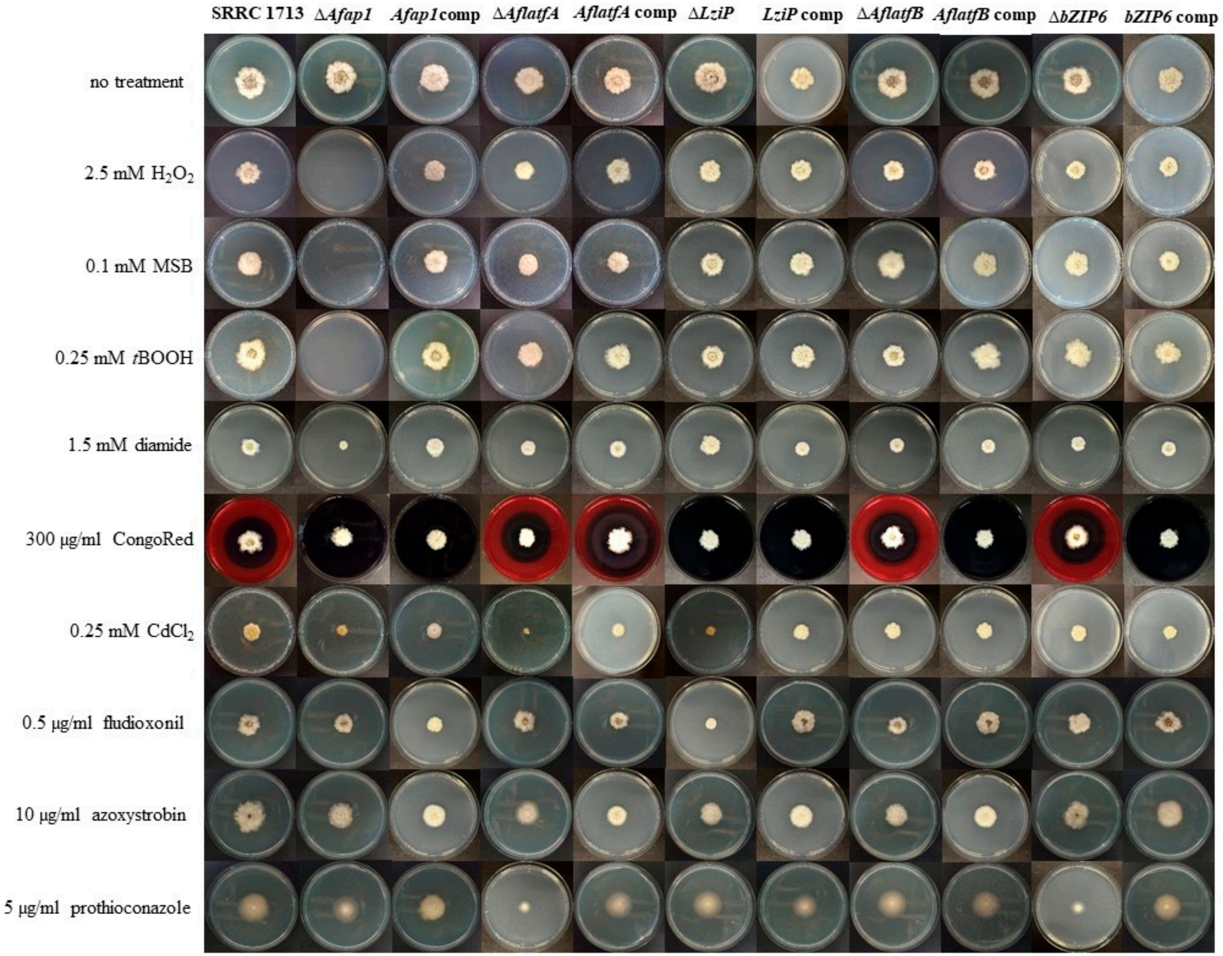

Figure S8. Stress sensitivity of the control and mutant strains. Stress sensitivities observed in surface cultures on GMM agar plates are shown. GMM agar plates were incubated at 30 °C for 5 days. Photos show representative colony growth of the strains.
