# Supplementary material for: Aspergillus flavus bZIP-Type Transcription Factors as Promising Novel Targets for Future Aflatoxin Control Strategies
Source: J Fungi (Basel). 2026 Jul 19;12(7):532. doi: 10.3390/jof12070532 (PMC13412632; doi:10.3390/jof12070532)
Supplement: Supplementary file 1 [file jof-12-00532-s001.zip › jof-4333994 Supplementary Tables S1 and S2_mod.pdf]

Table S1 *Aspergillus* strains used in this study

**Strains**	**Genotype**	**Reference**
<u>*Aspergillus flavus*</u>		
SRRC 1713	*Δku70, ΔniaD, ΔpyrG, ptrA*^{s}	[30]
Afap1	*Δku70, ΔniaD, ΔpyrG,* 🐦*Afap1::ptrA*	This study
AflatfA	*Δku70, ΔniaD, ΔpyrG,* 🐦*AflatfA::ptrA*	This study
LziP	*Δku70, ΔniaD, ΔpyrG,* 🐦*LziP::ptrA*	This study
AflatfB	*Δku70, ΔniaD, ΔpyrG,* 🐦*AflatfB::ptrA*	This study
bZIP6	*Δku70, ΔniaD, ΔpyrG,* 🐦*bZIP6::ptrA*	This study
Afap1 comp	*Δku70, ΔniaD, ptrA*^{R}, 🐦*Afap1::ptrA, Afap1+, AnipyrG+*	This study
AflatfA comp	*Δku70, ΔniaD, ptrA*^{R}, 🐦*AflatfA::ptrA, AflatfA+, AnipyrG+*	This study
LziP comp	*Δku70, ΔniaD, ptrA*^{R}, 🐦*LziP::ptrA, LziP+, AnipyrG+*	This study
AflatfB comp	*Δku70, ΔniaD, ptrA*^{R}, 🐦*AflatfB::ptrA, AflatfB +, AnipyrG+*	This study
bZIP6 comp	*Δku70, ΔniaD, ptrA*^{R}, 🐦*bZIP6::ptrA, bZIP6+, AnipyrG+*	This study
<u>*Aspergillus nidulans*</u>		
FGSC A4	wild type, veA^{+}	[90]
<u>Plasmid</u>		
pPTRI	*Aspergillus oryzae ptrA*	[91]

References

30. Cary, J.W.; Harris-Coward, P.; Scharfenstein, L.; Mack, B.M.; Chang, P.-K.; Wei, Q.; Lebar, M.; Carter-Wientjes, C.; Majumdar, R.; Mitra, C.; et al. The *Aspergillus flavus* homeobox gene, *hbx1*, is required for development and aflatoxin production. *Toxins* **2017**, *9*, 315.

90. McCluskey, K. The Fungal Genetics Stock Center, from molds to molecules. *Adv. Appl. Microbiol.* **2003**, *52*, 245–262.

91. Cary, J.W.; Han, Z.; Yin, Y.; Lohmar, J.M.; Shantappa, S.; Harris-Coward, P.Y.; Mack, B.; Ehrlich, K.C.; Wei, Q.; Arroyo-Manzanares, N.; et al. Transcriptome analysis of *Aspergillus flavus* reveals *veA*-dependent regulation of secondary metabolite gene clusters, including the novel aflavarin cluster. *Eukaryot. Cell* **2015**, *14*, 983–997.

Table S2 Oligonucleotides used in this study

Name of primer	Sequence of primer 5'-3'	Used for
ptrA-F	GGGCAATTGATTACGGGATCCC	5′*ptrA* marker
ptrA-R	CAAGAGCGGCTCATCGTCACCC	3′*ptrA* marker
Afap1 upstream fwd	CCTATTGCCACTGGTGCTCACGGC	5′-flanking region of *Afap1*
Afap1 upstream kim rev	GGGATCCCGTAATCAATTGCCCTCGTTCTATCGTATGAAGTCGCC	5′*Afap1* with *ptrA* tail
Afap1 downstream kim fwd	CAAGAGCGGCTCATCGTCAACTTTCATTTTGGGATCAACATTATCC AACGAT	3′*Afap1* with *ptrA* tail
Afap1 downstream rev	TCGTGACCTGTTCCCTGAGGAGGA	3′-flanking region of *Afap1*
Afap1 nested fwd	TACAGGGTACGCATTCTCTTGAGA	5′ nested of *Afap1*
Afap1 nested rev	TCAACAGTGTCGAAGAGGCCAAGG	3′ nested of *Afap1*
AflatfA upstream fwd	GTCAGGGATTGTACATCGTCAACTGGCCTGCA	5′-flanking region of *AflatfA*
AflatfA upstream kim rev	GGGATCCCGTAATCAATTGCCCTAATTTAGCTGGAGGTGACGGGT TATATTAGA	5′ *AflatfA* with *ptrA* tail
AflatfA downstream kim fwd	CAAGAGCGGCTCATCGTCACCCGAATTCAAAAATTGGACTGGCTG AATCTGATG	3′ *AflatfA* with *ptrA* tail
AflatfA downstream rev	ACAATCACGCTCATTAACCTCAGTGCCATTGT	3′-flanking region of *AflatfA*
AflatfA nested fwd	GGCAATCCAAGAAATTCGACTTGTCTTGTC	5′ nested of *AflatfA*
AflatfA nested rev	CAACGAAAGCCGACAACCCTTTATATAGCA	3′ nested of *AflatfA*
LziP upstream fwd	AGAACATCAGTTTTGCCTCAATTCCACGACAT	5′-flanking region of *LziP*

LziP upstream kim rev	GGGATCCCGTAATCAATTGCCCCCGGATGATAGCGAGAGACGAG GTGGAGGTCG	5′ *LziP* with *ptrA* tail
LziP downstream kim fwd	CAAGAGCGGCTCATCGTCACCCCCATTTAGGCAGTTCAGTTCCCTG AACCTGCG	3′ *LziP* with *ptrA* tail
LziP downstream rev	TGATATCGTGATTCCGCACAATATAGGAAACA	3′-flanking region of *LziP*
LziP nested fwd	GCATAAGTGGCTATGCCGTTGAACGGG	5′ nested of *LziP*
LziP nested rev	CGAATTGAACATGTCCGTTATAACCGACTGCC	3′ nested of *LziP*
AflatfB upstream fwd	TGGAGATCATGATACAACAATCGGATTA	5′-flanking region of *AflatfB*
AflatfB upstream kim rev	GGGATCCCGTAATCAATTGCCCATATGTCAGACCTTGGGGAGGCG AAGAG	5′ *AflatfB* with *ptrA* tail
AflatfB downstream kim fwd	CAAGAGCGGCTCATCGTCACCCGGTGTCGGATAAAAGCGACTTGC ATAAT	3′ *AflatfB* with *ptrA* tail
AflatfB downstream rev	AGTGGGAACTGGATCTCGGACCAATGGA	3′-flanking region of *AflatfB*
AflatfB nested fwd	GTTATGATTATTATGAATCGATTATCGT	5′ nested of *AflatfB*
AflatfB nested rev	ATGTTCAATACTTAGTGATCTAACTA	3′ nested of *AflatfB*
bZIP6 upstream fwd	AAGCCCCGCCACGGCCAAAGAGATGCAAC	5′-flanking region of *bZIP6*
bZIP6 upstream kim rev	GGGATCCCGTAATCAATTGCCCAGCCGAAATAAAGCAAATTTATG CCTTT	5′ *bZIP6* with *ptrA* tail
bZIP6 downstream kim fwd	CAAGAGCAGCTCATCGTCACCCGAGAAGAGATATAAGAAGAACG GAGGA	3′ *bZIP6* with *ptrA* tail
bZIP6 downstream rev	CCGATGTTATTTACAATCCTTCTGGACC	3′-flanking region of *bZIP6*

bZIP6 nested fwd	CTACCTACGTGACCAAGGCAGAGCGG	5′ nested of *bZIP6*
bZIP6 nested rev	CAACACTGTCCTGGGTAGTATCCACACGTT	3′ nested of *bZIP6*
AnipyrG fwd	CAGACAATGCTCTCTATCCT	5′ *AnipyrG* selection marker for the complementation casettes
AnipyrG rev	CCAACTCCAATCTTCAAGAC	3′ *AnipyrG* selection marker for the complementation casettes
Afap1 comp fwd	GATAGTGCACGTTCCCCTTGTGGTTAGTGC	5′ *Afap1* comp
Afap1 comp kim rev	AGGATAGAGAGCATTGTCTGTAGAGAGTAGAATAATTAATTCAATATCAA	3′ *Afap1* comp with *AnipyrG* tail
Afap1 comp nested fwd	CTCGTTGTAGTTAGAAGGTGATTGGGG	5′ nested *Afap1* comp
AnipyrG nested rev	GGAACCTGACTACTACAATGTTGGGGATTT	3′ nested *AnipyrG*
AflatfA comp fwd	GACGGGTGTCCGTTTTTGAGTTGGTCTGTC	5′ *AflatfA* comp
AflatfA comp kim rev	CAGGATAGAGAGCATTGTCTGGTAAAACAGCGCATGGTTTCCTGCCCCGAGTC	3′ *AflatfA* comp with *AnipyrG* tail
AflatfA comp nested fwd	TGCATAATTCGCTGCTTGATCGTGATTATT	5′ nested *AflatfA* comp
LziP comp fwd	GCATAAGTGGCTATGCCGTTGAACGGG	5′ *LziP* comp
LziP comp kim rev	AGGATAGAGAGCATTGTCTGCGTTTCCGCGAAAGGCTTTGAATA	3′ *LziP* comp with *AnipyrG* tail

LziP comp nested fwd	CCAGAATCCCCGCTAATGGTGGAC	5′ nested *LziP* comp
AflatfB comp fwd	GCATTCTCACAGTTCGATGGAGTCACTGA	5′ *AflatfB* comp
AflatfB comp kim rev	AGGATAGAGAGCATTGTCTGATGTTCAATACTTAGTGATCTAACTA	3′ *AflatfB* comp with *AnipyrG* tail
AflatfB comp nested fwd	CCTTGGAAGGTCTGGACACATGGTCC	5′ nested *AflatfB* comp
bZIP6 comp fwd	GCATCTGACGCCCAAGCTCCCAATCCAT	5′ *bZIP6* comp
bZIP6 comp kim rev	AGGATAGAGAGCATTGTCTGGTTTGGCGGTGATCATCATGTCCCGAT	3′ *bZIP6*c omp with *AnipyrG* tail
bZIP6 comp nested fwd	CTGGTCTACAATAACAATAGGCACTTTCC	5′ nested *bZIP6* comp
Afap1 fwd	ATGGCCGATTACAATACCCTCTA	5′ *Afap1*
Afap1 rev	TTATTTGACGCGACCCATGATA	3′ *Afap1*
AflatfA fwd	ATGGACGCGAAAAAGAACGCTTTGGAAA	5′ *AflatfA*
AflatfA rev	TCATTGTCTTCGGAGTCCCTGTGTAGG	3′ *AflatfA*
LziP fwd	ATGGCCACATTAGCGGAACACC	5′ *LziP*
LziP rev	TCATGAGAGATGAGAGGTGACCA	3′ *LziP*
AflatfB fwd	ATGTCGGTGGACCAAACC	5′ *AflatfB*
AflatfB rev	CTAAACATTAATCAGCTCTTCAAAACTGTC	3′ *AflatfB*
bZIP6 fwd	ATGGCTTCAATCACCGTGGTA	5′ *bZIP6*
bZIP6 rev	CTAGTAGCTAATCATCTCGAACAAAGT	3′ *bZIP6*
